# Self-reported knowledge about dental caries at young age and variations between dental practitioners in the Ministry of Health in Bahrain

**DOI:** 10.1038/s41405-021-00073-6

**Published:** 2021-05-13

**Authors:** Eman Flaihan Alrowaili

**Affiliations:** Oral and Dental Health Services, Ministry of Health, Manama, Kingdom of Bahrain

**Keywords:** Preventive dentistry, Dental public health, Dental education, Dental epidemiology

## Abstract

**Objectives:**

To assess basic knowledge on dental caries and its prevention especially in young children and variation thereof between dental practitioners working for the Ministry of Health in Bahrain.

**Materials and methods:**

Self-reported questionnaire was sent to all dentists and dental hygienists.

**Results:**

One hundred and thirty-four dental practitioners responded. The mean knowledge and practice scores for association between diet and dental caries was 64.9 ± 20.1. Females scored higher than males (*p* = 0.011) and dentists scored higher than dental hygienists (*p* = 0.009). A low mean score 45.5 ± 19.6 was observed in preventing dental caries in toddlers. Those >40 years of age and those with >15 years of experience have significantly higher scores than other groups (*p* = 0.009 and *p* = 0.001), respectively. Mean score for caring for children’s teeth, which covered advice on fluoridated toothpaste, was 63.4 ± 20.9. Younger dentists had higher score than those >40 years of age (*p* = 0.003). Mean score for frequency of fluoride varnish application was 64.4 ± 15.8 with no significant variation between practitioners.

**Conclusion:**

There is a need to reduce variations and update and improve dental practitioners’ knowledge in regard to risks and prevention of caries at young age.

## Introduction

Dental caries are the most common noncommunicable disease worldwide and it is caused by increased intake of free sugars. All ages are at risk of developing dental caries but children and adolescents are at higher risk and reducing this risk at young age is beneficial at later life.^1^

Dental caries are prevented through different strategies and methods, a part of which is the application of fluoride varnish^2,3^ and empowering people to take control over their health and making appropriate choices.^4^ This can be achieved through providing information to patients and caretakers on diet, drinking habits, oral hygiene measures, and using fluoridated toothpaste.^5^ To be effective, such advice should be consistent and should be based on the best currently available evidence.^6,7^ Nevertheless, several studies from different countries showed variation and a lack of knowledge on caries and its prevention among dentists^8–12^ and dental hygienists.^13–15^

The population in the Kingdom of Bahrain is young with around 25.9% in the age group of 0–19 years old.^16^ Dental and Oral Health Services in the Ministry of Health (MOH) provide a wide range of primary and secondary dental care free of cost to a large population and provide different oral health education programs.^17^ A recent unpublished survey that involved government schools’ students, showed that dental caries experience was seen in 86.8% of 6-year-old children, 56.4% in those who are 12 years old, and 59% in those who are 15 years old.^18^ Similar findings were reported in other gulf cooperation council states.^19,20^‏

The high prevalence of dental caries in Bahrain especially at young age warrants the need to evaluate the different aspects of caries prevention available to this age group. One of which, is the need to assess the knowledge of dental practitioners—working in the dental and oral health services in MOH—on dental caries in general and at young age in particular and assess the preventive messages provided to parents and caretakers and variations thereof. The study also aims at examining the frequency of using fluoride varnish in dental clinics since a formerly set fluoride varnish program is not available. This is the first study in the dental and oral health services to tackle these issues.

## Materials and methods

A cross-sectional design was carried in the Dental and Oral Health Services in MOH in Bahrain. A self-administered structured multi-choice questionnaire was designed to examine dental practitioners’ knowledge in several oral health prevention-related issues. Different resources were reviewed to aid in formulating and adapting the questionnaire such as similar studies, Cochrane reviews, WHO guidelines, and other accepted guidelines and guidance, especially Delivering better oral health: an evidence-based toolkit for prevention, Department of Health, UK.^6^

The questionnaire comprised of 50 questions but in this paper, the scales that will be discussed are those related to association between diet and dental caries, preventing dental caries in toddlers, caring for children’s teeth, and the use of fluoride varnish. All in total are 14 questions in Likert scale and no open questions (Table 1). The questionnaire was checked for face validity with an expert and piloted for comprehensibility among three dentists and one dental hygienist not included in the sample. A minor change in the wording of one question was made. The study was approved by Primary Care Research Committee in the MOH.

**Table 1:** Statements and questions of the questionnaire with suggested and appropriate responses.

Scale	Statement/question	Suggested responses				
		1	2	3	4	5
Relation between diet and dental caries	The increase in intake of sugars causes dental caries	Yes^a^	No	I do not know		
	Sweetened juices cause dental caries	Yes^a^	No	I do not know		
	Fruits can cause dental caries	Yes	No^a^	I do not know		
	All types of carbohydrates can cause dental caries	Yes	No^a^	I do not know		
Preventing dental caries in toddlers	Breastfeeding (mother’s milk) can cause dental caries	Yes	No^a^	I do not know		
	Bottle milk formula can cause dental caries	Yes^a^	No	I do not know		
	It is advised to start giving children liquids by an open cup at the age of …	6 months^a^	12 months (1 year)	18 months	2 years	I do not know
	At which age a child should stop using feeding bottle?	6 months	12 months (1 year)^a^	18 months	2 years	I do not know
Caring for children’s teeth	How often do you ask parents about the type of toothpaste they use for their children?	Always^a^	Very often^a^	Sometimes	Rarely	Never
	What type of toothpaste do you usually advise for children?	Toothpastes that show age range	Any children’s toothpaste	Regular toothpaste (otherwise known as adult/family toothpaste)^a^	Herbal toothpaste	I do not usually specify type of toothpaste
	How often do you advise parents/caretakers on the amount of toothpaste to be used for their children?	Always^a^	Very often^a^	Sometimes	Rarely	Never
	Is there an age until which parental supervision on child’s brushing is needed?	Yes^a^	No	I do not know		
	Do you think brushing without a toothpaste can prevent dental caries?	Yes	No^a^	I do not know		
Preventing and managing dental caries in clinic	In regard Fluoride varnish, how often do you place it as a caries prevention and management measure?	Very often^a^	Occasionally	Rarely	Never	

^a^Appropriate response.

Total number of all dental hygienists, dentists, and dental specialists working in the primary and secondary services were requested from the Dental and Oral Health Services and the Oral Health Training Services in the MOH. All members of the targeted population were included in the study; 200 dental professionals in total (61 dental hygienists and 139 dentists).

Due to the Covid-19 situation in the Kingdom of Bahrain and abiding by the regulations of the MOH, the health practitioners were all contacted through smart phone application and a link to the questionnaire was sent with an explanatory message and contact details of the researcher in case of questions. The participants were informed if they choose to participate in the survey to answer all questions and submit their response. The link allows for anonymous replies and eliminates the possibility of missing responses. Answering and submitting the questionnaire were taken as a positive consent. The link was sent in June 2020 and a reminder was sent again after 10 days to improve response rate.

### Statistical analysis

SPSS 23 was used for data entry and analysis. Frequencies and percentages were computed for the categorical variables. Means and standard deviations were computed for quantitative variables. Regarding the knowledge and practice scoring, a score of one was assigned for correct knowledge or practice response and a score of zero was assigned to false knowledge or practice response and hence the higher the score, the greater level of knowledge or practice. The total score was computed for each scale for every participant. Then the total score was standardized by being divided by the number of questions in the scale and multiplied by 100. Finally, the mean and standard deviation of standardized scores of the participants were computed. The standardized means and standard deviations of the knowledge and practice scores were computed in relation to the demographical variables. Chi-Squared test and Fisher’s Exact test were used to determine association between two categorical variables. *T*-test and ANOVA were used to determine difference in standardized mean scores as appropriate. Post Hoc tests were used to investigate pairwise comparisons. In all statistical tests, *p* value of <0.05 was considered statistically significant.

## Results

Out of 200 dental professionals (61 dental hygienists and 139 dentists), 134 responded (41 dental hygienists and 93 dentists out of which 14 are specialists) with a total response rate of 67%. The majority of the dental staff are Bahrainis 127 (94.8%) and only 7 (5.2%) non-Bahraini specialists out of which 2 are pediatric dentists. Females make the majority in the sample 76.9%. The respondents are young, with mean age 33.1 ± 8.3 years and those who are <40 years of age constitute 80.6% of the sample. The mean years of experience since graduation is 9.0 ± 7.3 and the highest proportion 46.3% have <6 years of experience (Table 2). Most of the dental practitioners in the MOH are females and their current retirement age is 55 years, this could explain the sample’s distribution.

**Table 2:** Demographic characteristics (total = 134) and relationship between demographic characteristics and knowledge score of association between diet and dental caries.

	*n* (%)	Association between diet and dental caries Mean ± SD	*p* value
Age in years			
≤30	75 (56)	62 ± 20.3	0.115
31–40	33 (24.6)	66.7 ± 16.1	
>40	26 (19.4)	71.2 ± 23.1	
Gender			
Female	103 (76.9)	67 ± 21.1	**0.011**
Male	31 (23.1)	58.1 ± 15	
Qualification			
Dental hygienist	41 (30.6)	58.5 ± 17.3	**0.009**
Dentists	93 (69.4)	67.7 ± 20.7	
Years of experience since graduation			
≤5	62 (46.3)	62.5 ± 19.1	0.139
6–10	29 (21.6)	62.1 ± 21.8	
11–15	19 (14.2)	67.1 ± 14.6	
>15	24 (17.9)	72.9 ± 23.2	

*T*-test showed a significant mean difference between female and male with *p* value = 0.011 and 95% CI (2.1, 15.7) and between dental hygienists and dentists with *p* value = 0.009 and 95% CI (−16.1, −2.3).

Statistically significant *p*-values are in bold.

### Association between diet and dental caries

The questions in this scale assessed dental practitioners’ knowledge on free sugars. The overall standardized mean knowledge score of this scale (out of 100) for all dental practitioners was 64.9 (SD ± 20.1). Most of the respondents identified that the increase intake of sugars causes caries and that sweetened juices cause dental caries. Only around a third did not think fruits cause caries and a similar proportion correctly responded that not all carbohydrates cause dental caries (Table 3). Significant variation between respondents was seen in the gender and qualifications subgroups (Table 2).

**Table 3:** Percentages of correct and false responses on questions of different scales.

	False	Correct
	*n* (%)	*n* (%)
The increase in intake of sugars causes dental caries	6 (4.5)	128 (95.5)
Sweetened juices cause dental caries	11 (8.2)	123 (91.8)
Fruits can cause dental caries	87 (64.9)	47 (35.1)
All types of carbohydrates can cause dental caries	84 (62.7)	50 (37.3)
Breastfeeding (mother’s milk) can cause dental caries	89 (66.4)	45 (33.6)
Bottle milk formula can cause dental caries	4 (3)	130 (97)
At what age is it advised to start giving children liquids by an open cup	98 (73.1)	36 (26.9)
At which age a child should stop using feeding bottle?	101 (75.4)	33 (24.6)
How often do you ask parents about the type of toothpaste they use for their children?	34 (25.4)	100 (74.6)
What type of toothpaste do you usually advise for children?	107 (79.9)	27 (20.1)
How often do you advise parents/caretakers on the amount of toothpaste to be used for their children?	17 (12.7)	117 (87.3)
Is there an age until which parental supervision on child’s brushing is needed?	22 (16.4)	112 (83.6)
Do you think brushing without a toothpaste can prevent dental caries?	65 (48.5)	69 (51.5%)

### Preventing dental caries in toddlers

The questions in this scale examined dental practitioners’ knowledge on relation between dental caries and child feeding methods, and knowledge on when to start using open cup and when to wean infants of feeding bottles when used.

The standardized mean knowledge score of this scale (out of 100) for the dental practitioners was the lowest among all scales; 45.5 (SD ± 19.6). The question concerning formula milk as a risk factor for dental caries at young age was the only question with a high correct response rate, all other questions had low correct response rate (Table 3).

Significant difference between standardized mean scores was seen within age subgroups and years of experience (Table 4). Dental professionals who are more than 40 years of age had comparatively higher knowledge scores (55.8 ± 24.8) than younger practitioners (*p* = 0.009) and those who had more than 15 years of experience had comparatively higher standardized mean knowledge score (58.3 ± 27.53) than those with <10 years of experience (*p* = 0.001).

**Table 4:** Relationship between demographic characteristics and knowledge score of preventing dental caries in toddlers and knowledge score of caring for children’s teeth.

	Preventing dental caries in toddlers Mean ± SD	*p* value	Caring for children’s teeth Mean ± SD	*p* value
Age in years				
≤30	42.3 ± 15.9	**0.009**	64.3 ± 16.9	**0.003**
31–40	44.7 ± 20.5		70.3 ± 24.6	
>40	55.8 ± 24.8		52.3 ± 22.7	
Gender				
Female	45.6 ± 21.1	0.884	62.5 ± 21.1	0.361
Male	45.2 ± 13.6		66.5 ± 20.3	
Qualification				
Dental hygienist	44.5 ± 18.1	0.693	65.9 ± 15.6	0.307
Dentist	46 ± 20.3		62.4 ± 22.8	
Years of experience since graduation				
≤5	42.3 ± 16.1	**0.001**	64.8 ± 18.4	0.172
6–10	39.7 ± 15.7		64.8 ± 17.4	
11–15	48.7 ± 17.6		67.4 ± 26.8	
>15	58.3 ± 27.3		55 ± 24.5	

LSD showed a significant mean difference between age group ≤30 and age group >40 with *p* value = 0.002 and 95% CI (−22.0, −4.9) and between age group 31–40 and age group >40 with *p* value = 0.028 and 95% CI (−21.0, −1.2). Also, between experience group ≤5 and experience group >15 with *p* value = 0.001 and 95% CI (−24.9, −7.1) and between experience group 6–10 and experience group >15 with *p* value < 0.001 and 95% CI (−28.9, −8.5). The test also showed a significant mean difference between age group ≤30 and age group >40 with *p* value = 0.010 and 95% CI (2.9, 21.0) and between age group 31–40 and age group >40 with *p* value = 0.001 and 95% CI (7.5, 28.4).

### Caring for children’s teeth

This scale covered the advice provided to parents and caretakers for preventing caries at home through brushing with fluoridated toothpaste. The standardized mean knowledge score out of 100 in this scale for the respondents was 63.4 (SD ± 20.9).

Almost half 69 (51.5%) think brushing without a toothpaste does not prevent dental caries. The respondents who always/very often inquired about the type of toothpaste used for children at home were 100 (74.6%) but only 27 (20.1%) advised the correct type of toothpaste (regular or adult toothpaste for children). The majority of respondents 117 (87.3%) always and very often advised parents on the toothpaste amount to be used for their children and 112 (83.6%) recognized the need for parental supervision on child’s brushing (Table 3).

Significant variation in knowledge and practice scores was only seen between age subgroups, with the younger practitioners showing comparatively better scores than those who are more than 40 years of age (Table 4).

### Using fluoride varnish for preventing and managing dental caries in clinic

The standardized mean score for the frequency of using fluoride varnish in preventing caries by all dental practitioners was 64.4 (SD ± 15.8). Sixty-six (49.3%) of dental practitioners applied fluoride varnish very often, 62 (46.3%) occasionally and six (4.5%) rarely. No significant differences were seen between practitioners in using fluoride varnish for caries prevention (Table 5).

**Table 5:** Relationship between demographic characteristics and placing fluoride varnish for caries prevention and management.

	Fluoride varnish application		*p* value
	Rarely/occasionally	Very often	
	*n* (%)	*n* (%)	
Age in years			
≤30	32 (42.7)	43 (57.3)	0.058
31–40	18 (54.5)	15 (45.5)	
>40	18 (69.2)	8 (30.8)	
Gender			
Female	50 (48.5)	53 (51.5)	0.353
Male	18 (58.1)	13 (41.9)	
Qualification			
Dental hygienist	20 (48.8)	21 (51.2)	0.445
Dentists	48 (51.6)	45 (48.4)	
Years of experience since graduation			
≤5	25 (40.3)	37 (59.7)	0.119
6–10	16 (55.2)	13 (44.8)	
11–15	13 (68.4)	6 (31.6)	
>15	14 (58.3)	10 (41.7)	

## Discussion

The study focuses on dental practitioners’ core knowledge and variation thereof on dental caries and its prevention especially at young age. This is the first study to assess these issues in Bahrain MOH dental practitioners.

The overall result showed that dental practitioners in general have reasonable standardized mean score knowledge and practice related to caries, except in the scale that measured (Risk and association of dental caries at young age). In addition, variations in knowledge and practice were seen between dental practitioners especially when assessed against age.

### Association between diet and dental caries

Free sugars added to foods are implicated in the cause of dental caries and sweetened beverages are their primary source.^1^ Also, staple starchy foods and fruits have not been proven to cause dental caries.^1,21^ The exact term (free sugars) was not used in this survey, to correctly assess practitioners’ knowledge on the foods and drinks that contain free sugars. Dental practitioners’ knowledge about the relation between dental caries and sugars in general and sweetened juices was found to be good but updating is needed on fruits and carbohydrates.

The majority of dental practitioners 95.5% recognized sugars in general as the cause of caries, this is higher than the 80% reported by Lin et al.^10^ In regard to sweetened juices; 91.8% of respondents recognized them as a cause of dental caries. The term (fresh juices) was not used in the questionnaire as it might have been confusing since one of the daily five portions of fruits and vegetables can be 150 ml of unsweetened fresh juice.^6^ Confusion regarding fresh juices is thought to be seen in a study that assessed dental students’ knowledge on diet in which 73.0% related fresh juices to dental caries.^22^

Significant variations in this scale were seen, females scored higher than males (67 ± 21.1 Vs 58.1 ± 15: P = 0.011), which was also seen in other studies that assessed caries knowledge and prevention.^23,24^

Variation between dentists and dental hygienist was only seen in this scale. Dentists scored comparatively higher than dental hygienists (67.7 ± 20.7 vs. 58.5 ± 17.3 *p* = 0.009). Francisco et al.^14^ and Manski and Parker^15^ assessed dental hygienists knowledge on caries and management and concluded that improvement in the same was needed. This difference between practitioners in this score specifically may indicate that even though dental hygienists’ knowledge in this sample is comparable to that of dentists in relation to risk of caries at young age, their knowledge on fluoridated toothpaste and the use of fluoride varnish, yet updating their knowledge on free sugars, foods that contain them and WHO guidelines is needed.

### Preventing dental caries in toddlers

The least standardized mean knowledge score and the most variation in the answers were seen in this scale. The question with maximum number of correct response (97%) was for the association between milk formula and dental caries. The practitioners were clear about this information as none of them chose (I do not know) option. But, respondents’ knowledge on breast milk needs updating. Multiple recent studies have refuted the old ambiguous relation between breast milk and dental caries.^25–27^ Not many studies assessed dental practitioners’ knowledge on breastfeeding but a study that involved young dentists in East Midlands found that 81% lacked knowledge about it.^28^

Dental practitioners’ knowledge about when to start giving liquids by open cup and when to stop using feeding bottle was also low. A recent Cochrane review suggested that advice to pregnant women and those with children <1 year of age leads to a reduced risk in early childhood caries.^29^

A significant difference in knowledge was seen between age groups and years of experience. Those who are more than 40 years of age and those who have more than 15 years of experience had comparatively higher mean scores than those who are younger and with less years of experience. This comes in contrast to other studies^24,30^ that showed younger dentists were more likely to provide better knowledge and management for children with dental caries. On the other hand, a study by Pakdaman et al.^31^ reported that younger respondents had better management and knowledge related to dental caries, but their youngest subgroups included those who are 35–44 years of age, which overlaps with the elder age group in our sample.

### Caring for children’s teeth

A Cochrane reviews concluded that fluoridated toothpastes containing above 1000 part per million of fluoride are effective in preventing caries^32^ and a dose response effect is observed in children and adolescents.^33^ The use of fluoridated toothpastes is one of the reasons for dental caries decline in western countries.^34^ In Norway, a Scandinavian country with low caries levels^35^ 84–98% of dental and other health professionals recommended fluoridated toothpaste to all children.^11^ For maximum caries control, it was advised that all children should use toothpastes that contain 1350–1500 ppm fluoride with parental supervision,^6^ such toothpastes are commonly known in our community as adult or regular toothpastes and hence this term was used in the questionnaire.

No clear information is currently available about the levels of fluoride in domestic water and for that matter if water purification is used widely at homes in our population. Henceforth, a need arises for the use of optimal fluoride concentration in toothpastes.

Dental practitioners’ knowledge was acceptable in certain aspects and lacking in others in regard to fluoridated toothpastes as a method of caries prevention. Most respondents recognized the importance of parental supervision, inquired about the toothpaste type parents’ use for their children and advised them on toothpaste amount. Nevertheless, 98 (73.1%) of respondents advised suboptimal fluoride toothpastes, 1 (0.7%) advised herbal toothpastes that do not contain fluoride, and 8 (6%) did not advise parents on type of toothpaste to use for their children. Only 20% advised the correct type, which is lower than the 48.3% reported by Yusuf et al.^24^ This is worrying because it may indicate that children are not exposed enough to fluoride.

Another worrying finding was that almost half of the respondents (48.5%) thought brushing alone—without toothpaste—can prevent caries, a low response was reported by Ghasemi et al.^8^ where the mean score of respondents thinking fluoridated toothpaste more important than brushing was (1.11 ± 1.09), four being the maximum score. A slightly better finding was reported by Lin et al.^10^ where only 29.2% thought brushing without toothpaste is effective in children. The same study, however, reported that when the respondents were asked to rate first and second effective methods in preventing caries, only 8% chose fluoridated toothpastes while 24% and 16%, respectively, chose prophylaxis and flossing.

In regard to variation, significant difference in scores was seen between age groups, where younger practitioners scored better than those who are more than 40 years of age, similar findings were reported elsewhere.^24,30^

### Using fluoride varnish for preventing and managing dental caries in clinic

Currently, no formally set fluoride application program is available in the MOH and hence, the use of fluoride varnish for caries prevention depends on practitioners.

Only this question had a 4 point Likert scale responses (very often, occasionally, rarely, and never) but all the responses were aggregated in the first three options and none of the participants chose (never). This could indicate practitioners’ propensity toward prevention.

The proportion of dental practitioners who very often apply fluoride varnish for preventing caries is 49.3%, which is low considering the prevalence of caries in the population. In comparison, it is similar to the 43.4% reported by Lin et al.^10^ but <52.1% reported by Yusuf et al.^24^ Of dental hygienists, 51.2% very often provided fluoride varnish, which in comparison is higher than the 25% reported by Maryland dental hygienists.^15^ Even though no significant variation was seen between practitioners but in comparison young, females, with <5 years of experience applied fluoride varnish more often than others. Yusuf et al.^24^ reported a similar finding but with significant difference.

The study overall shows the need to improve and update dental practitioners’ knowledge about free sugars, causes and risks, and association of dental caries, its prevention at young age especially related to the use of fluoridated toothpastes, and the need to increase fluoride varnish application for preventing caries. In addition, there is a need to reduce variations between dental practitioners’ to prevent possible contradictory advice provided to patients and parents.

Along with continuous education—and even though several international guidelines are available—there is a need to develop local oral health prevention guidance/guidelines that are adapted to the population’s disease levels, and services provided. Taking into account that dental health massages as advised by Kowash et al.^36^ should be cultural sensitive, consider the socio-economic status of parents and provide them with acceptable alternatives.

Several studies called for improving the teaching of prevention at college level for dentists^8^ and dental hygienists.^15^ There is no dental school in Bahrain but dental hygienists teaching program is available and updated information need to be incorporated in their curricula.

The study is limited by only covering oral health practitioners working in the MOH while dental care is provided by other government facilities, private clinics, and hospitals. The study is descriptive and suffers from known limitations of self-reported knowledge and practice studies such as response bias and inflated figures in practice questions. Moreover, the study only assessed the basic knowledge of dental practitioners on dental caries and prevention for children. However, it is the first to tackle this issue in the MOH Dental and Oral Health Services. More qualitative and quantitative studies are needed to assess in depth the knowledge, practice, and attitude of dental practitioners toward prevention for different age groups.

## Conclusion

In general, the basic knowledge of dental practitioners working for the MOH about dental caries can be considered reasonable, but there is a need for improvement and updating especially in relation to knowledge and prevention of caries in young children. There is also a need to decrease the variations seen between dental practitioners to aid in providing consistent and evidence-based advice to parents and caretakers to help them make better choices and aid in preventing caries in children.
